# Variation in the Stable Carbon and Nitrogen Isotope Composition of Plants and Soil along a Precipitation Gradient in Northern China

**DOI:** 10.1371/journal.pone.0051894

**Published:** 2012-12-18

**Authors:** Jian-Ying Ma, Wei Sun, Xiao-Ning Liu, Fa-Hu Chen

**Affiliations:** 1 Cold and Arid Regions Environmental and Engineering Research Institute, Chinese Academy of Sciences, Lanzhou, People’s Republic of China; 2 Key Laboratory of Western China’s Environmental Systems (Ministry of Education), Lanzhou University, Lanzhou, People’s Republic of China; 3 Institute of Grassland Science, Key Laboratory of Vegetation Ecology (Ministry of Education), Northeast Normal University, Changchun, People’s Republic of China; Jyväskylä University, Finland

## Abstract

Water availability is the most influential factor affecting plant carbon (*δ* ^13^C) and nitrogen (*δ* ^15^N) isotope composition in arid and semi-arid environments. However, there are potential differences among locations and/or species in the sensitivity of plant *δ* ^13^C and *δ* ^15^N to variation in precipitation, which are important for using stable isotope signatures to extract paleo-vegetation and paleo-climate information. We measured *δ* ^13^C and *δ* ^15^N of plant and soil organic matter (SOM) samples collected from 64 locations across a precipitation gradient with an isotherm in northern China. *δ* ^13^C and *δ* ^15^N for both C_3_ and C_4_ plants decreased significantly with increasing mean annual precipitation (MAP). The sensitivity of *δ* ^13^C to MAP in C_3_ plants (-0.6±0.07‰/100 mm) was twice as high as that in C_4_ plants (−0.3±0.08‰/100 mm). Species differences in the sensitivity of plant *δ* ^13^C and *δ* ^15^N to MAP were not observed among three main dominant plants. SOM became depleted in ^13^C with increasing MAP, while no significant correlations existed between *δ* ^15^N of SOM and MAP. We conclude that water availability is the primary environmental factor controlling the variability of plant *δ* ^13^C and *δ* ^15^N and soil *δ* ^13^C in the studied arid and semi-arid regions. Carbon isotope composition is useful for tracing environmental precipitation changes. Plant nitrogen isotope composition can reflect relative openness of ecosystem nitrogen cycling.

## Introduction

In drought-prone ecosystems, water availability controls ecosystem structure and processes by affecting long-term balances between ecosystem inputs and outputs of elements and the cycling of carbon and nutrients within ecosystems [1]. The effects of water availability on nutrient cycling in ecosystems are complex. Studies along natural gradients of water availability are helpful and can address these controls [2]. Plant performance along environmental gradients offers one way to evaluate potential plant responses to climate change [3]. Stable carbon and nitrogen isotopic signatures (*δ* ^13^C and *δ* ^15^N) of plants and soil can serve as valuable non-radioactive tracers and nondestructive integrators of how plants today and in the past have integrated with and responded to their abiotic and biotic environments [4], [5], [6].

Plants discriminate against ^13^CO_2_ during photosynthetic CO_2_ fixation in ways that reflect plant metabolism and environmental conditions. Differences between carboxylation reactions induce the disparate photosynthetic ^13^C fractionation and response to changes in environmental conditions between the C_3_ and C_4_ photosynthetic pathways [7]. Carbon isotopic composition is affected by the ratio of ambient and intercellular humidities and should therefore reflect changes in the energy budgets of leaves, which are themselves influenced by stomatal conductance [8]. C_3_ plants growing under water-stressed conditions are expected to be enriched in ^13^C compared to plants growing under optimal water conditions [8]. Indeed, negative correlations between mean annual precipitation (MAP) and *δ* ^13^C value of C_3_ plants have been demonstrated in a number of studies [5], [9], [10], [11]. In contrast to C_3_ plants, the *δ* ^13^C values of C_4_ plants are expected to be less sensitive to water stress [12]. Accordingly, no correlation between the *δ* ^13^C values of C_4_ plants and water availability (e.g. precipitation) is commonly observed [5], [10], [13].

The ^13^C/^12^C ratios of soil organic matter (SOM) are influenced by both the relative abundance and *δ* ^13^C values of C_3_ and C_4_ species as plants are the primary C sources of SOM. Therefore, the *δ* ^13^C values of SOM in loess and paleosols can be used to extract paleoclimate and associated vegetation composition information [10], [14]. However, paleovegetation reconstruction using *δ* ^13^C of SOM could introduce errors without correction for the effects of precipitation on plant *δ* ^13^C [10].

Plant and soil nitrogen isotopic composition (*δ* ^15^N) is related to the environmental variables and availability of nutrients and water; therefore, it can be used as an indicator of ecosystem N cycling on different spatial and temporal scales [6], [15]. The changes in *δ* ^15^N values in both soils and plants along natural precipitation gradients can be used to identify the pattern of nitrogen losses relative to turnover among these sites [2]. An enrichment of ^15^N in soil and plant samples has been demonstrated for precipitation gradients within the arid desert environments [16], [17], [18]. Following rain events, processes that cause the loss of N discriminate against the heavier ^15^N isotope, favoring larger proportional loss of ^14^N and increasing *δ* ^15^N of the remaining N in ecosystems [19]. Handley et al. [17] proposed that the observed negative correlations between plant *δ* ^15^N values and precipitation are a product of water availability and soil N sources during plant growth. As a result of difference in mycorrhizal association and presence or absence of N_2_-fixing symbiosis, species-dependent sensitivity of *δ* ^15^N to variation in MAP may confound correlations between plant *δ* ^15^N values and precipitation [5], [13], [20], [21]. For example, *δ* ^15^N in N_2_-fixing plant is expected to be not sensitive to changes in precipitation [21], [22].

**Figure 1:**
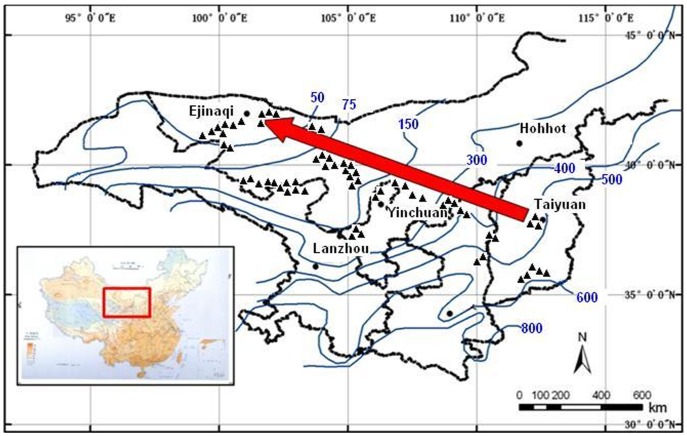
Location of study area in China. The thin solid dark blue lines are isolines of mean annual precipitation. Triangles are sampling sites.

Water availability, measured as rainfall, is argued to be the most influential factor affecting plant *δ* ^13^C and *δ* ^15^N in semi-arid and arid environments [5], [11], [15], [21]. The relationships between rainfall and plant C and N isotopic composition have been demonstrated in many regions, but the sensitivity of plant C and N isotopic composition to variation in precipitation varies significantly among different locations and/or different species composition [10]. In this study, in order to eliminate the temperature influence and focus on precipitation effect, we examined large-scale patterns in *δ* ^13^C and *δ* ^15^N of plant and soil organic matter across a regional precipitation gradient along an isotherm with mean annual temperature of 8°C in northern China (Fig. 1). Questions addressed include: what is the response of C and N isotopic signatures in both plant and soil to a precipitation gradient in northern China; how do C_3_ and C_4_ plants differ in their response to changes in precipitation; and whether there are species specific differences in the response of stable carbon and nitrogen isotopic signatures to the precipitation gradient.

## Results

### Plant Carbon and Nitrogen Stable Isotope Composition

**Figure 2:**
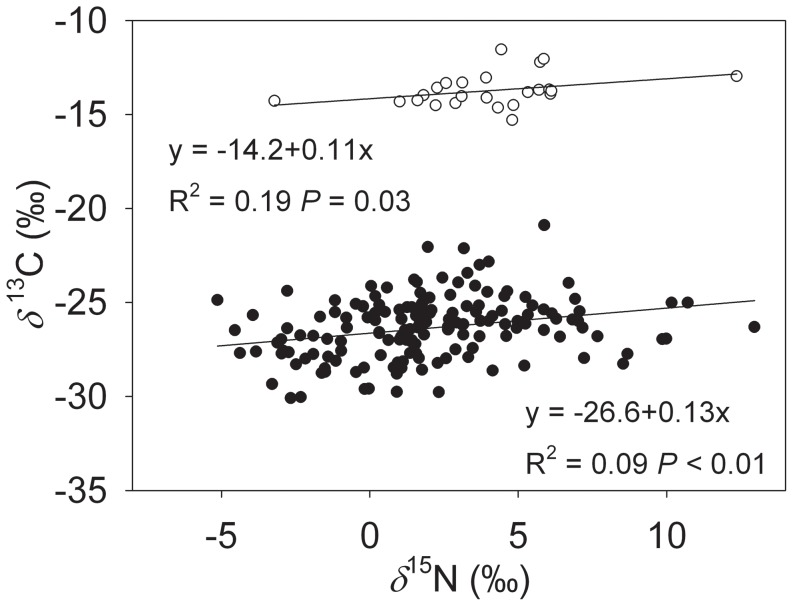
Correlations between *δ* ^13^C and *δ* ^15^N values of the studied C_3_ (filled circles) and C_4_ plants (open circles). Linear regression equations, *R* ^2^ and *P* values are provided.

The *δ* ^13^C values of all samples ranged from −31.1‰ to −11.6‰ and fell into two distinct groups. The *δ* ^13^C values of C_3_ plants varied from −31.1‰ to −20.9‰, while the *δ* ^13^C values of C_4_ plants had a much narrower range from −15.3‰ to −11.6‰ (Table S1). The *δ* ^15^N values of C_3_ and C_4_ plants ranged from −5.1‰ to 13.0‰ and from −3.2‰ to 12.4‰, respectively (Table S1). Although plant *δ* ^13^C and *δ* ^15^N values were significantly correlated, the model explained very little of the variation (Fig. 2; *R* ^2^<0.2, *P*<0.05). There were no significant differences between C_3_ and C_4_ photosynthetic pathways in the slope of linear correlation (Table 1; *P* = 0.45).

**Table 1:** Degrees of freedom (*df*), F and *P* values from slope comparison analysis to assess differences in sensitivity between C_3_ and C_4_ species, as well as among the three studied shrubs: *Nitraria sibirica* (NS), *Reaumuria soongorica* (RS) and *Hedysarum mongolicum* (HM).

		*df*	F	*P*
Sensitivity of *δ* ^ 13^C to MAP	C_3_ vs C_4_	1	7.78	<0.01
	NS vs RS	1	0.91	0.35
	NS vs HM	1	1.39	0.25
	RS vs HM	1	0.46	0.50
Sensitivity of *δ* ^ 15^N to MAP	NS vs RS	1	0.88	0.35
	NS vs HM	1	2.05	0.16
	RS vs HM	1	0.37	0.55
Correlation between *δ* ^ 13^C and *δ* ^ 15^N	C_3_ vs C_4_	1	0.72	0.45

### Correlations between Mean Annual Precipitation and Plant Stable Isotope Composition

**Figure 3:**
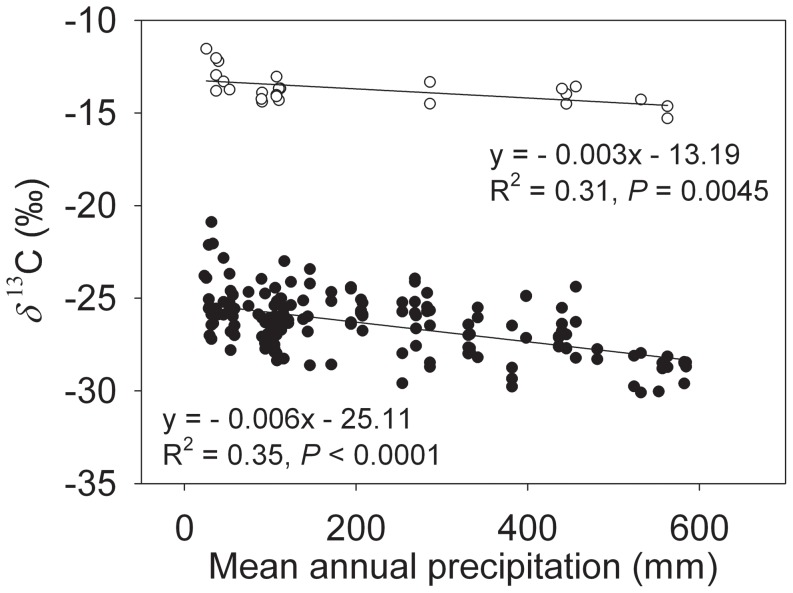
Plant *δ* ^13^C values (C_3_, filled circles; C_4_, open circles) as a function of mean annual precipitation. Linear regression equations, *R* ^2^ and *P* values are provided.

Significant negative correlations were found between plant *δ* ^13^C values and mean annual precipitation in both C_3_ (Fig. 3; *R* ^2^ = 0.35, *P*<0.01) and C_4_ (Fig. 3; *R* ^2^ = 0.31, *P*<0.01) plants. The regression slope of *δ* ^13^C to precipitation (Table 1; *P*<0.01) in C_3_ plants (−0.6±0.07‰/100 mm) was significantly greater than that of C_4_ plants (−0.3±0.08‰/100 mm).

**Figure 4:**
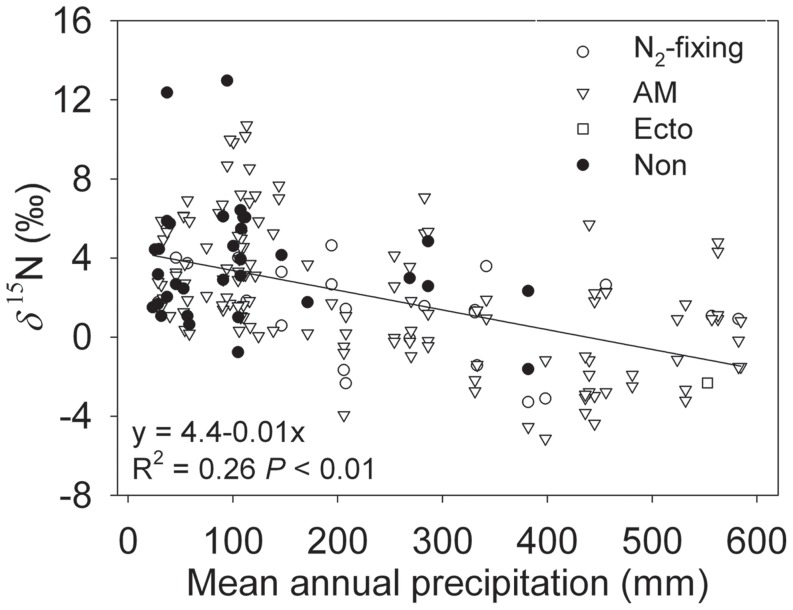
Plant *δ* ^15^N values (N_2_-fixing, open circles; AM, open triangles; Ecto, open squares; Non, filled circles) as a function of mean annual precipitation. Linear regression equations, *R* ^2^ and *P* values are provided.

Plant *δ* ^15^N showed a significantly negative correlation with precipitation (Fig. 4; *R* ^2^ = 0.26, *P*<0.01). The regression slope of *δ* ^15^N to precipitation is −1.0±0.1‰/100 mm.

**Figure 5:**
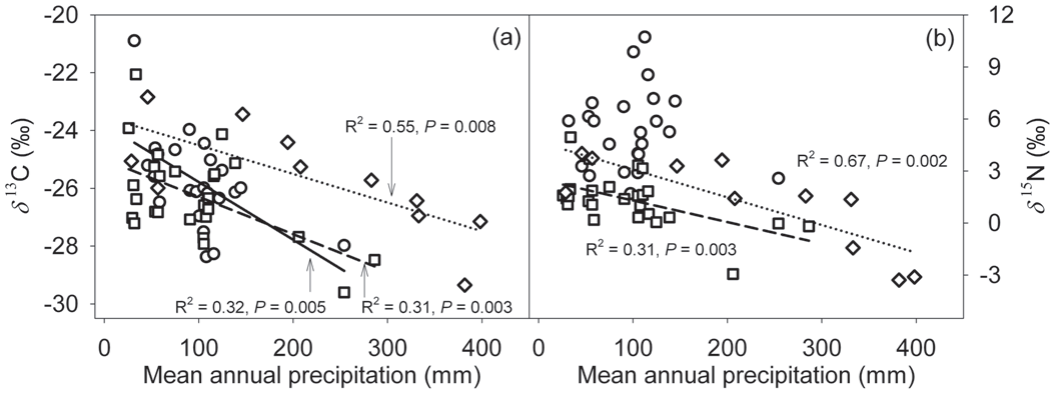
Leaf δ^13^C (a) and δ^15^N (b) values as a function of mean annual precipitation in *Nitraria sibirica* (circle, solid line), *Reaumuria soongorica* (square, dash line) and *Hedysarum mongolicum* (diamond, dotted line). R^2^ and P values of linear correlations are provided.

The *δ* ^13^C value of three dominant C_3_ species was correlated negatively with the amount of precipitation (Fig. 5a). There were no differences among *Nitraria sibirica* Pall., *Reaumuria soongorica* (Pall.) Maxim. and *Hedysarum mongolicum* Turcz. in the sensitivity of leaf *δ* ^13^C and *δ* ^15^N to variation in precipitation (Table 1; *P*>0.05). The *δ* ^15^N values of *H*. *mongolicum* (Fig. 5b; *R* ^2^ = 0.67, *P* = 0.002) and *R. soongarica* (Fig. 5b; *R* ^2^ = 0.31, *P* = 0.003) were significantly negatively correlated with mean annual precipitation. No significant correlation existed between *δ* ^15^N values of *N. sibirica* (Fig. 5b; *R* ^2^ = 0.002, *P* = 0.83) and mean annual precipitation.

### Correlations between Mean Annual Precipitation and the Carbon and Nitrogen Isotope Composition of Soil Organic Matter

**Figure 6:**
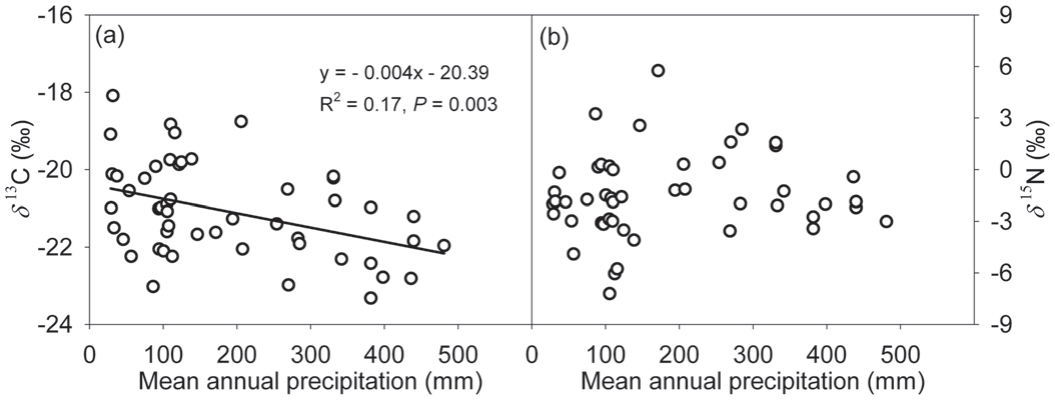
Soil organic matter *δ* ^13^C (a) and *δ* ^15^N (b) values as a function of mean annual precipitation. Linear regression equations, *R* ^2^ and *P* values are provided.

The *δ* ^13^C and *δ* ^15^N values of soil organic matter were plotted against mean annual precipitation in Fig. 6. Soil organic matter *δ* ^13^C and *δ* ^15^N tended to decrease with increasing mean annual precipitation, but only the relationship between the *δ* ^13^C values of soil organic matter and precipitation was significant (Fig. 6a; *R* ^2^ = 0.17, *P* = 0.003). The response of soil *δ* ^13^C to precipitation amount is −0.4±0.1‰/100 mm for the precipitation range of 25–600 mm. No significant correlations existed between *δ* ^15^N values of soil organic matter and mean annual precipitation (Fig. 6b; *R* ^2^ = 0.012, *P* = 0.45).

## Discussion

### Carbon Isotopes

Plants balance their needs between CO_2_ intake for photosynthesis and conservation of water by adjusting the conductance of their leaf stomata. An increase in precipitation (water availability) would result in an increase in the stomatal conductance that in turn causes a decrease in the plant *δ* ^13^C value [10], [12]. For C_3_ species, significant negative correlation between plant *δ* ^13^C and water availability, indicated by precipitation, has been observed in many regions [3], [9], [10], [11], [23], [24], [25], [26]. Similarly, we observed that plant *δ* ^13^C correlated negatively with MAP across a rainfall gradient ranging from 25 mm to 600 mm in northern China. The sensitivity of *δ* ^13^C response of C_3_ plants to annual precipitation (−0.6±0.07‰/100 mm) in our study was comparable with that reported for the Chinese Loess Plateau (−0.7‰/100 mm) [10]. Negative correlations between plant *δ* ^13^C and MAP may have resulted from water availability associated variation in photosynthetic discrimination.

Some uncertainties still exist in the correlation between *δ* ^13^C of C_4_ plants and environmental factors. Depending on how much CO_2_ and HCO_3_^-^ in bundle sheath cells leak out into mesophyll cells (*φ*,leakiness), the response of C_4_ photosynthetic carbon isotope discrimination to precipitation can be positive, zero or negative [27]. Positive correlations between C_4_ photosynthetic carbon isotope discrimination and precipitation suggest *φ* values above 0.34, as *φ* will affect the discrimination of Rubisco against ^13^C [28], [29]. In southern Africa, the *δ* ^13^C values of C_4_ plants are not sensitive to changes in the MAP [5]. Van de Water et al. [3] reported a significant decrease of *δ* ^13^C value in a C_4_ species *Atriplex confertifolia* with elevation (precipitation increase with elevation) in the Southwest United States. Wang et al [30] found that *δ* ^13^C of C_4_ plants in the dry season was lower than in the wet season, which suggests that there is a positive correlation between *δ* ^13^C of C_4_ plants and precipitation in the Loess Plateau of China. We observed that *δ* ^13^C value of C_4_ plants was negatively correlated with MAP (−0.3±0.08‰/100 mm), which is comparable to the results of Liu et al (−0.43‰/100 mm) [10]. Positive correlations between C_4_ photosynthetic carbon isotope discrimination and precipitation suggest leakiness values above 0.34 [28], [29], which were likely given the studied C_4_ plants are growing in water-limited areas [31]. Further studies are needed to determine the importance of leakiness in determining the response of *δ* ^13^C of C_4_ plants to environmental factors. The regression slope of *δ* ^13^C of C_4_ plants (−0.3±0.08‰/100 mm) on precipitation was much lower than that of C_3_ plants (−0.6±0.07‰/100 mm), which suggests that *δ* ^13^C of C_4_ plants is less sensitive to variation in environmental water availability than that of C_3_ plants.

The sensitivity of leaf *δ* ^13^C to changes in water availability also varies substantially among locations or C_3_ species. In eastern Australia, leaf *δ* ^13^C of C_3_ species exhibited significant negative correlation with precipitation from 300 to 1700 mm [9], while in northern Australia, the response of plant *δ* ^13^C to precipitation was shown only within a precipitation range from 200 to 450 mm, whereas average plant *δ* ^13^C of sites remained constant between 450 and 1800 mm precipitation [13]. In addition, Miller et al [25] studied a series of co-occurring and replacement *Eucalyptus* species along a rainfall gradient in Australia, suggesting leaf carbon isotope discrimination in five of 13 species decreased with decreasing rainfall, seven exhibited no trend, and one increased. We found no differences in the sensitivity of leaf *δ* ^13^C to variation in precipitation among the three desert shrubs, 2 nonN_2_-fixing plants (*N. sibirica* and *R*. *soongorica*) and a legume shrub *H*. *mongolicum.* The observed sensitivity of leaf *δ* ^13^C to MAP in the three shrubs was slightly higher than the result (−1.1‰/100 mm) of a study conducted in arid northwest China [10]. The inconsistency may have resulted from both differences in sampling area and plant life forms. Liu et al. [10] collected the grass species from the precipitation range of 200–700 mm while we sampled desert shrubs within a precipitation range from 25 to 600 mm. Similar, or even slightly higher sensitivity of leaf *δ* ^13^C to MAP between desert shrubs and grasses suggest desert shrubs are also very sensitive to changes in water availability.

Community *δ* ^13^C is a successful empirical predictor of water availability within the usual range of C_3_ whole-leaf *δ* ^13^C values. In this context the *δ* ^13^C signature can be used as an indicator of the environmental influences over plant function, especially at the community level.

The *δ* ^13^C value of soil organic matter reflects the relatively long-term isotopic composition of the standing biomass [14], [32]. The *δ* ^13^C of the soil organic matter significantly increased from −23.3‰ in the southeast to −18‰ in the northwest along the declining precipitation gradient (Fig. 6a), which may have resulted from both increased plant *δ* ^13^C values with decreasing precipitation for the examined C_3_ and C_4_ species and enhanced contribution of C_4_ plants to soil organic carbon at the drier sites (Fig. 3).

### Nitrogen Isotopes

The observed mean leaf nitrogen isotope values are comparable to the published data set collected from the Loess Plateau of China [33] and Mount Kinabalu, Borneo [34]. The range of variation in leaf *δ* ^15^N (−5.1‰ to 13.0‰) (Table S1) is greater than that in Chinese Loess Plateau [33] and Mount Kinabalu [34], however the observed shifts in leaf *δ* ^15^N are within the range of foliar *δ* ^15^N (from >−10‰ to <15‰) reported by Craine et al. [21]. The observed large variation in leaf *δ* ^15^N is possible given that we sampled multiple plant species across a broad range of climate and ecosystem types.

The observed negative correlations between plant *δ* ^15^N and precipitation (Fig. 4) are in agreement with the results of previous studies [5], [11], [15], [17], [18], [33]. However, there are differences in the sensitivity of plant *δ* ^15^N to variation in mean annual precipitation. In southern Africa, nitrogen isotope composition of C_3_ plants was significantly correlated with mean annual precipitation, with plant *δ* ^15^N values declining 0.68‰ with every 100 mm increased in precipitation [5]. Data from Zambia, Namibia and South Africa indicated that plant *δ* ^15^N values declined 0.47‰ with every 100 mm increase in precipitation [18]. We observed a 1.0±0.1‰ decrease in plant *δ* ^15^N values with every 100 mm increase in precipitation, which is greater than the results of those studies conducted in Africa, but similar to the results obtained in the Chinese Loess Plateau [33].

Plant *δ* ^15^N is related to the availability of nutrients and water; therefore, it is an indicator of N cycling on both spatial and temporal scales [5]. Under high N availability conditions, isotopically depleted N is preferentially lost from the ecosystem through the processes of NH_3_ volatilization, denitrification and leaching of NO_3_^−^, which results in an enrichment of soil N pools in ^15^N and subsequent increases in leaf *δ* ^15^N. Conversely, plants growing under low N availability conditions are likely to depend on mycorrhizal fungi for N acquisition than at high N availability, plant N obtained via mycorrhhizal fungi is depleted in ^15^N [35], [36]. The observed increase in plant and soil *δ* ^15^N with decreasing MAP suggests the arid and semi-arid regions are more open in terms of their N cycling relative to those that are more humid [5], with higher N losses relative to turnover [2].

Plant *δ* ^15^N values are determined by the availability, distribution and isotopic signature of soil N sources, preferential uptake of isotopcially different N compounds, plant metabolic processes involved N fractionation, especially the formation of mycorrhizal symbiosis [19], [36], [37]. Plants fix C directly from the atmosphere, while they obtain N from soil or through a symbiotic relationship with N-fixing microorganisms [11], so soil processes can play an important role in plant isotopic signatures [17]. In a synthesis study, Craine et al. [21] observed that non-mycorrhizal plants are enriched in ^15^N relative to species having mycorrhizal symbiosis. Moreover, plant *δ* ^15^N differed among mycorrhizal types, with *δ* ^15^N in arbuscular mycorrhizal plants greater than ectomycorrhizal plants. In our study, we observed that the average *δ* ^15^N in non-mycorrhizal plants (3.6‰) is greater than mycorrhizal plants (1.9‰), which is in agreement with the result of Craine et al. [21]. Lower *δ* ^15^N values in mycorrhizal plants suggests mycorrhizal fungi create ^15^N-depleted compounds that are subsequently transferred to host plants [36].

The *δ* ^15^N values of *H. mongolicum* (−1.6±0.4‰/100 mm; *R* ^2^ = 0.67, *P* = 0.002) showed the most significant correlation and the steepest regression slope across the precipitation gradient than that of the other two species *R. soongarica* (−1.2±0.4‰/100 mm; *R* ^2^ = 0.31, *P* = 0.003) and *N. sibirica* (−0.3±1.1‰/100 mm; *R* ^2^ = 0.002, *P* = 0.827), which is contrary to our prediction. Legume species obtain their N through symbiotic N_2_-fixing bacteria, therefore the *δ* ^15^N of N_2_-fixing plants might be independent of climate and not reflect soil processes [21], [22]. However, the observed significant correlation between leaf *δ* ^15^N and MAP in legume species *H. mongolicum* suggests potential shift in the reliance of legume species on N_2_-fixing bacteria as N source in high nitrogen availability habitats.

We observed no significant correlation between *δ* ^15^N values of soil organic matter and mean annual precipitation, which is inconsistent with the results of previous studies [15], [18], [33]. Strong negative correlations of *δ* ^15^N values of soil organic matter and mean annual precipitation have been observed in the Chinese Loess Plateau (1.31‰/100 mm) [33] and in the Kalahari region of southern Africa (0.56‰/100 mm) [18]. In general, *δ* ^15^N values of soils and plants depleted with increasing precipitation suggests that accumulated losses of nitrogen relative to pools are greater in the drier sites. Nitrogen cycling is more open in drier sites and becomes less open with increasing precipitation [2]. Although N cycling on a regional scale involves numerous and complex processes, our study showed that spatial variability of precipitation play a significant role on isotopic signatures and the N cycle in the soil-plant system [18].

The correlation between precipitation gradient and community-averaged plant C and N isotope values provide insights into the cycling of terrestrial N and water status of plants in response to climatic change. Given that plant isotope value is a biological expression of environmental conditions integrated over time, it may indeed provide us a more meaningful measure of water availability than rainfall data [9]. In this respect, we can argue that the *δ* ^13^C and *δ* ^15^N of plants might be used as an indicator of environmental influences on plant functioning, and further evaluate how plants respond to their habitats.

In conclusion, along the precipitation gradient with an isotherm in northern China, *δ* ^13^C and *δ* ^15^N values of C_3_ and C_4_ plants were significantly negatively correlated with MAP. The *δ* ^13^C values of C_3_ plants are more sensitive to variation in MAP than *δ* ^13^C values of C_4_ plants. There were no species differences in the sensitivity of plant *δ* ^13^C and *δ* ^15^N to MAP among three dominant species *H. mongolicum*, *R. soongarica* and *N. sibirica*. The *δ* ^13^C values of soil organic matter became significantly more depleted with increasing MAP, while no significant correlations existed between *δ* ^15^N values of soil organic matter and MAP. We concluded that water availability is the primary environmental factor controlling the variability of plant *δ* ^13^C and *δ* ^15^N and soil *δ* ^13^C in the arid and semi-arid regions. Water-limited systems in northern China are more open in terms of nutrient cycling compared to those that have adequate water supply and therefore the resulting natural abundance of foliar ^15^N in these systems is enriched.

## Materials and Methods

### Study Area

The study area is located in northern China with latitudes ranging from 35°36′ to 42°54′, and longitudes from 99°25′ to 113°42′ (Fig. 1). The climate of the study area is temperate arid and semi-arid. The dominant control over the amount of precipitation is the strength of East Asian monsoon system, which is mostly accompanied with cool, dry winters and hot, wet summers, with most of the rain falling in the summer season [38]. From southeast to northwest of the study area, the amount of annual rainfall decreases from 600 mm to 25 mm along an isotherm of 8°C. The meteorological data were obtained from the Cold and Arid Regions Environmental and Engineering Research Institute, Chinese Academy of Sciences. The vegetation is dominated by shrubs and grasses of both C_3_ and C_4_ plants in this region. In general, the vegetation of the study area changes progressively from forest steppe, dry steppe to desert steppe with decreasing precipitation [39].

### Field Sampling

In September 2006, leaf and soil samples were collected from 64 sites along the southeast to northwest precipitation gradient with an isotherm (Fig. 1). Detailed information of the sampling sites, including location, vegetation and precipitation is provided in Table S1. In each sampling site, fully expanded leaves of each dominant species were collected from three different individuals 5 m apart from each other and pooled into one sample. During the sampling period, most of the sampled plant species were at their late growing stage. Leaf samples were air-dried in the field, rinsed and oven-dried to a constant weight at 60°C in the laboratory, and finely ground with a ball mill. During the field campaign, 576 individuals of 31 dominant species were collected. Soil samples at a depth of 2–3 cm were collected using a corer (wiping off the superficial soil in 0.5 cm depth) from each of the 64 sampling sites. For each sampling site, three soil samples (each has a volume about 100 ml) were collected and pooled into one sample. The soil samples were passed through a 2 mm sieve to remove roots and gravels. Subsamples of the sieved soil were ground to a fine powder in a mortar and pestle, acidified in 6N HCl to remove coexisting carbonate, rinsed in deionized H_2_O, and dried through lyophilization [40]. Ground leaf samples and pre-treated soil samples were measured on a mass spectrometer for stable isotope composition analysis (described below). Sampling sites were selected from undisturbed land to avoid potential effects of anthropogenic activities on plant and soil *δ* ^13^C and *δ* ^15^N values.

No specific permits were required for the described field studies. No specific permissions were required for the use of sampling locations and collecting of soil and leaf samples because the sampling locations are not privately-owned or protected in any way and the field studies did not involve endangered or protected species.

### Isotopic Analysis

*δ* ^13^C and *δ* ^15^N analysis were done using a Finnigan Delta Plus XP continuous flow inlet isotope ratio mass spectrometer attached to a Costech EA 1108 Element Analyzer at the University of Wyoming Stable Isotope Facility. Precision of repeated measurements of laboratory standard was <0.1‰. *δ* ^13^C values are reported relative to V-PDB and *δ* ^15^N to AIR in parts per thousand (‰) as:
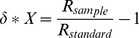
where 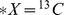 or 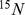 and 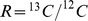 or 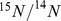 [41].

### Statistical Analyses

Simple linear regression analyses were used to estimate relationships between mean annual precipitation and *δ* ^13^C and *δ* ^15^N values of leaf and soil. All statistical analyses were carried out using SAS version 9.0 (SAS Institute Inc. Cary, NC, USA).

## Supporting Information

Table S1 Sample sites information (Location, Altitude, Mean annual precipitation, Vegetation type and collected species) being presented.
(DOC)
