# Supplementary material for: Variation in the Stable Carbon and Nitrogen Isotope Composition of Plants and Soil along a Precipitation Gradient in Northern China
Source: PLoS One. 2012 Dec 18;7(12):e51894. doi: 10.1371/journal.pone.0051894 (PMC3525597; doi:10.1371/journal.pone.0051894)
Supplement: Table S1 — Sample sites information (Location, Altitude, Mean annual precipitation, Vegetation type and collected species) being presented. (DOC) [file pone.0051894.s001.doc]

**Supplementary Table 1**. Sample sites information (Location, Altitude, Mean annual precipitation, Vegetation type and collected species) being presented.

| Sample site | Location | Altitude (m) | Mean annual precipitation (mm) | Vegetation type | Collected species | N_2_-fixing | Mycorrhizal types^*^ | *δ*^13^C (‰) | *δ*^15^N (‰) |  |
| --- | --- | --- | --- | --- | --- | --- | --- | --- | --- | --- |
| Sanchaxiang, Shanxi | 35°36´N  112°42´E | 1100 | 584.6 | Shrubs | *Ziziphus jujuba var.spinosa* (Bunge)Hu  *Artemisia vulgaris* L. | No  No | AM  AM | -28.47  -28.7 | 0.81  -1.5 |  |
| humenggou, Shanxi | 35°37´N  112°50´E | 1056 | 582.4 | Shrubs | *Artemisia annua* L.  *Artemisia vulgaris* L.  *Caragana microphylla* Laxm. | No  No  Yes | AM  AM  AM | -29.61  -28.51  -28.45 | -0.17  -1.51  0.91 |  |
| Heshui, Shanxi | 35°57´N  113°04´E | 1100 | 562.7 | Shrubs | *Artemisia annua L.*  *Artemisia vulgaris* L.  *Bothriochloa ischaemum* (L.) Keng  *Achnatherum splendens*(Trin.) Nevski | No  No  No  No | AM  AM  AM  AM | -28.73  -28.15  -14.65  -15.3 | 0.92  1.12  4.32  4.8 |  |
| Taibai, Shanxi | 36°07´N  113°42´E | 1090 | 556.9 | Shrubs | *Caragana microphylla* Laxm.  *Artemisia vulgaris* L. | Yes  No | AM  AM | -28.5  -28.8 | 1.07  0.91 |  |
| Ziwuling, Shanxi | 36°05´N  113°21´E | 1320 | 552.7 | Forest | *Pinus tabulaeformis* Carr. | No | Ecto | -30.04 | -2.32 |  |
| Jiyizhen, Shaanxi | 36°50´N  110°28´E | 631 | 531.9 | Shrubs | *Artemisia annua L.*  *Ziziphus jujuba var.spinosa* (Bunge)Hu  *Setaria viridis* (L.) Beauv. | No  No  No | AM  AM  AM | -30.1  -27.97  -14.29 | -2.66  1.66  -3.21 |  |
| Xiahuaxiang, Shaanxi | 36°48´N  110°36´E | 794 | 523.9 | Shrubs | *Artemisia annua L.*  *Ziziphus jujuba var.spinosa* (Bunge)Hu | No  No | AM  AM | -28.12  -29.77 | -1.14  0.91 |  |
| Xuecunzheng, Shanxi | 37°25´N  110°46´E | 828 | 481 | Grassland | *Artemisia roxburghiana* Bess.  *Artemisia vulgaris* L. | No  No | AM  AM | -27.76  -28.3 | -1.9  -2.48 |  |
| Suide, Shaanxi | 37°30´N  110°34´E | 1100 | 456 | Shrubs | *Artemisia annua L.*  *Caragana microphylla* Laxm.  *Ziziphus jujuba var.spinosa* (Bunge)Hu  *Chloris virgata* Swartz | No  Yes  No  No | AM  AM  AM  AM | -28.23 -26.29  -24.39  -13.59 | 2.29  2.64  -2.78  2.27 |  |
| Yizhou, Shanxi | 38°25´N  112°34´E | 900 | 445.1 | Shrubs | *Artemisia annua L.*  *Ziziphus jujuba var.spinosa* (Bunge)Hu  *Artemisia roxburghiana* Bess.  *Setaria viridis* (L.) Beauv.  *Achnatherum splendens*(Trin.) Nevski | No  No  No  No  No | AM  AM  AM  AM  AM | -27.7  -27.73  -26.97  -13.98  -14.52 | -4.37  -2.97  -2.98  1.81  2.22 |  |
| Yangqu, Shanxi | 38°13´N  112°45´E | 1060 | 439.9 | Shrubs | *Artemisia roxburghiana* Bess.  *Ziziphus jujuba var.spinosa* (Bunge)Hu  *Elymus dahuricus* Turcz.  *Bothriochloa ischaemum* (L.) Keng | No  No  No  No | AM  AM  AM  AM | -25.53  -26.78  -26.39  -13.7 | -1.17  -1.9  -2.78  5.7 |  |
| Wangshixiang, Shanxi | 38°11´N  112°29´E | 1350 | 436.2 | Shrubs | *Artemisia anethoides* Mattf.  *Roegntria nutans* (Keng) Keng  *Elymus dahuricus* Turcz.  *Artemisia tournefortiana* Reichb. | No  No  No  No | AM  AM  AM  AM | -27.12  -27.59  -27.07  -27.62 | -3.07  -2.91  -0.97  -3.83 |  |
| Changhan, Shaanxi | 38°23´N  109°38´E | 1150 | 398.2 | dune vegetation | *Hedysarum mongolicum* Turcz.  *Salix psammophila* C.Y.Wang et Ch.Y. Yang  *Elymus dahuricus* Turcz. | Yes  No  No | AM  AM  AM | -27.15  -24.88  -24.9 | -3.11  -5.13  -1.17 |  |
| Mahezhen, Shaanxi | 38°30´N  109°27´E | 1220 | 381.8 | dune vegetation | *Hedysarum mongolicum* Turcz.  *Salix psammophila* C.Y.Wang et Ch.Y. Yang  *Salsola ruthenica* Iljin.  *Bassia dasyphylla* (Fisch. et C.A.Mey.) | Yes  No  No  No | AM  AM  Non  Non | -29.35  -26.49  -28.76  -29.78 | -3.29  -4.54  -1.62  2.33 |  |
| Wushenqi, Neimeng | 38°43´N  109°09´E | 1310 | 341.9 | dune vegetation | *Caragana microphylla* Laxm.  *Agropyron cristatum* (L.) Gaertn  *Salix psammophila* C.Y.Wang et Ch.Y. Yang | Yes  No  No | AM  AM  AM | -25.51  -26.05  -28.2 | 3.58  1.9  0.95 |  |
| Bayanwenduer, Neimeng | 38°37´N  108°46´E | 1340 | 333 | dune vegetation | *Hedysarum mongolicum* Turcz.  *Agropyron cristatum* (L.) Gaertn  *Artemisia arenaria* DC. | Yes  No  No | AM  AM  AM | -26.95  -27.7  -27.9 | -1.43  1.36  -1.4 |  |
| Zhanggaiaobao, Neimeng | 38°47´N  108°32´E | 1360 | 331 | dune vegetation | *Salix psammophila* C.Y.Wang et Ch.Y. Yang  *Hedysarum mongolicum* Turcz.  *Hedysarum scoparium* Fisch. Et Mey.  *Artemisia arenaria* DC. | No  Yes  Yes  No | AM  AM  AM  AM | -27.66  -26.43  -27  -28 | -2.73  1.36  1.24  -2.17 |  |
| Sumitu, Neimeng | 38°56´N  108°15´E | 1410 | 282.9 | dune vegetation | *Hedysarum mongolicum* Turcz.  *Artemisia arenaria* DC.  *Artemisia roxburghiana* Bess. | Yes  No  No | AM  AM  AM | -25.72  -24.72  -25.48 | 1.56  5.25  7.07 |  |
| Etuokeqizhongxuchang, Neimeng | 39°08´N  107°55´E | 1500 | 270 | dune vegetation | *Artemisia roxburghiana* Bess.  *Roegntria nutans* (Keng) Keng  *Agropyron cristatum* (L.) Gaertn | No  No  No | AM  AM  AM | -27.58  -26.64  -25.94 | -0.96  0.32  1.83 |  |
| Etuokeqi, Neimeng | 39°15´N  107°30´E | 1360 | 207.8 | dune vegetation | *Hedysarum mongolicum* Turcz.  *Artemisia roxburghiana* Bess.  *Agropyron cristatum* (L.) Gaertn  *Sophora alopecuroides* L. | Yes  No  No  Yes | AM  AM  AM  AM | -25.26  -25.96  -25.88  -26.76 | 1.42  0.19  1.07  -2.34 |  |
| Qipanjingzhen, Neimeng | 39°18´N  107°08´E | 1360 | 171.4 | Desert | *Agropyron cristatum* (L.) Gaertn  *Roegntria nutans* (Keng) Keng  *Caragana jubata* (Pall.) Poir. Var. recurva Lion. | No  No  Yes | AM  AM  Non | -25.17  -24.67  -28.59 | 3.68  0.19  1.76 |  |
| Jilantai1, Neimeng | 39°19´N  105°56´E | 1350 | 146.5 | Desert | *Hedysarum mongolicum* Turcz.  *Hedysarum scoparium* Fisch. Et Mey.  *Zygophyllum gobicum* Maxim. | Yes  Yes  No | AM  AM  Non | -23.44  -24.22  -28.63 | 3.29  0.58  4.14 |  |
| Jilantai2, Neimeng | 39°22´N  105°42´E | 1150 | 143.9 | Desert | *Nitraria sibirica* Pall.  *Peganum harmala* L. | No  No | AM  AM | -25.99  -26.81 | 7.02  7.67 |  |
| Jilantai3, Neimeng | 39°41´N  105°43´E | 1000 | 86.5 | Desert | *Nitraria sibirica* Pall. | No | AM | -25.88 | 6.28 |  |
| Jilantaijinsanjiao, Neimeng | 39°30´N  105°36´E | 1070 | 113.2 | Desert | *Nitraria sibirica* Pall.  *Zygophyllum gobicum* Maxim. | No  Yes | AM  AM | -25.02  -26.72 | 10.71  1.83 |  |
| Jilantaibangbugetata, Neimeng | 39°35´N  105°17´E | 1230 | 115.9 | Desert | *Nitraria sibirica* Pall.  *Reaumuria soongorica* (Pall.) Maxim.  *Peganum harmala* L. | No  No  No | AM  AM  AM | -28.27  -25.58  -25.92 | 8.54  1.83  6.83 |  |
| Suhaitu1, Neimeng | 39°47´N  105°04´E | 1440 | 112 | Desert | *Salsola passerina* Bge.  *Reaumuria soongorica* (Pall.) Maxim.  *Peganum harmala* L.  *Lycium ruthenicum* Murr. | No  No  No  No | Non  AM  AM  AM | -13.69  -26.72  -25.02  -26.17 | 6.05  3.15  10.17  3.15 |  |
| Suhaitu2, Neimeng | 39°58´N  104°54´E | 1580 | 110.2 | Desert | *Lycium ruthenicum* Murr.  *Peganum harmala* L.  *Salsola passerina* Bge.  *Calligonum mongolicum* Trucz.  *Reaumuria soongorica* (Pall.) Maxim. | No  No  No  No  No | AM  AM  Non  AM | -26.17  -26.72  -13.69  -14.32  -26.34 | 4.56  3.15  6.05  1.01  1.62 |  |
| Suhaitu3, Neimeng | 40°05´N  104°53´E | 1390 | 108.1 | Desert | *Reaumuria soongorica* (Pall.) Maxim.  *Nitraria sibirica* Pall.  *Agropyron cristatum* (L.) Gaertn  *Ephedra przewalskii* Stapf. | No  No  No  No | AM  AM  AM  Non | -26.83  -28.37  -26  -25.17 | 1.06  5.21  4.98  5.48 |  |
| Sudier, Neimeng | 40°21´N  104°45´E | 1280 | 100.5 | Desert | *Nitraria sibirica* Pall.  *Ephedra przewalskii* Stapf. | No  No | AM  Non | -26.96  -26.8 | 9.86  4.6 |  |
| WulijiEast, Neimeng | 40°30´N  104°39´E | 1290 | 97.3 | Desert | *Nitraria sibirica* Pall.  *Lycium ruthenicum* Murr. | No  No | AM  AM | -26.12  -26.94 | 1.69  9.99 |  |
| Wuliji, Neimeng | 40°52´N  104°27´E | 1480 | 94.3 | Desert | *Lycium ruthenicum* Murr.  *Agropyron cristatum* (L.) Gaertn | No  No | AM  AM | -24.76  -27.44 | 2.01  3.47 |  |
| Suhongtu, Neimeng | 41°09´N  104°14´E | 933 | 90.8 | Desert | *Salsola passerina* Bge.  *Nitraria sibirica* Pall.  *Reaumuria soongorica* (Pall.) Maxim.  *Halostachys caspica* (M. Bieb.) C.A.Mey | No  No  No  No | Non  AM  AM  Non | -14.41  -26.08  -27.08  -13.91 | 2.89  2.92  1.39  6.09 |  |
| SuhongtuNorth, Neimeng | 41°18´N  103°53´E | 848 | 75 | Desert | *Nitraria sibirica* Pall.  *Reaumuria soongorica* (Pall.) Maxim. | No  No | AM  AM | -24.67  -25.42 | 4.54  2.08 |  |
| Delige, Neimeng | 41°29´N  103°28´E | 905 | 54.2 | Desert | *Nitraria sibirica* Pall.  *Reaumuria soongorica* (Pall.) Maxim.  *Agropyron cristatum* (L.) Gaertn | No  No  No | AM  AM  AM | -24.61  -26.81  -27.81 | 2.72  3.7  0.37 |  |
| YaganWest, Neimeng | 42°54´N  102°43´E | 1080 | 40 | Desert | *Reaumuria soongorica* (Pall.) Maxim.  *Salsola passerina* Bge. | No  No | AM  Non | -25.89  -12.22 | 1.07  5.74 |  |
| Huhetuolegai, Neimeng | 41°57´N  102°16´E | 1114 | 29.6 | Desert | *Reaumuria soongorica* (Pall.) Maxim  *Ephedra przewalskii* Stapf.. | No  No | AM  Non | -27.02  -25.46 | 1.42  4.45 |  |
| Wulanailigen, Neimeng | 42°00´N  101°44´E | 930 | 33.9 | Desert | *Reaumuria soongorica* (Pall.) Maxim. | No | AM | -26.37 | 4.95 |  |
| Ejinaqi1, Neimeng | 41°57´N  100°55´E | 948 | 37.3 | Desert | *Ephedra przewalskii* Stapf.  *Salsola collina* Pall.  *Calligonum mongolicum* Trucz.  *Halostachys caspica* (M. Bieb.) C.A.Mey | No  No  No  No | Non  Non  AM  Non | -25.57  -12.05  -13.82  -12.97 | 2.03  5.86  5.33  12.36 |  |
| Ejinaqi2, Neimeng | 41°49´N  100°33´E | 978 | 31.8 | Desert | *Reaumuria soongorica* (Pall.) Maxim.  *Nitraria sibirica* Pall.  *Ephedra przewalskii* Stapf.  *Artemisia arenaria* DC. | No  No  No  No | AM  AM  Non  AM | -27.21  -20.9  -25.89  -26.45 | 1.54  5.88  1.07  2.64 |  |
| Ejinaqi3, Neimeng | 41°47´N  100°24´E | 945 | 28.7 | Desert | *Tamarix ramosissima* Ledeb.  *Ephedra przewalskii* Stapf.  *Hedysarum mongolicum* Turcz.  *Kalidium cuspidatum* (Ung.-Sternb.) Grub. | No  No  Yes  No | AM  Non  AM  Non | -25.57  -22.13  -25.06  -25.55 | 2.79  3.17  1.76  1.67 |  |
| Ejinaqi4, Neimeng | 41°44´N  100°07´E | 973 | 23.7 | Desert | *Ephedra przewalskii* Stapf. | No | Non | -23.8 | 1.5 |  |
| Ejinaqi5, Neimeng | 41°36´N  99°53´E | 1010 | 25.8 | Desert | *Reaumuria soongorica* (Pall.) Maxim.  *Haloxylon ammodendron* (C.A.Mey.) Bge. | No  No | AM  Non | -23.92  -11.55 | 1.59  4.43 |  |
| Ejinaqi6, Neimeng | 41°26´N  99°43´E | 1080 | 33.4 | Desert | *Reaumuria soongorica* (Pall.) Maxim. | No | AM | -22.06 | 1.96 |  |
| Ejinaqi7, Neimeng | 41°14´N  99°27´E | 1370 | 45.9 | Desert | *Hedysarum mongolicum* Turcz.  *Nitraria sibirica* Pall.  *Ephedra przewalskii* Stapf.  *Calligonum mongolicum* Turcz. | Yes  No  No  No | AM  AM  Non  AM | -22.84  -25.21  -25.9  -13.31 | 4.01  3.27  2.67  3.12 |  |
| Ejinaqi8, Neimeng | 40°59´N  99°25´E | 1300 | 53 | Desert | *Reaumuria soongorica* (Pall.) Maxim.  *Nitraria sibirica* Pall.  *Ephedra przewalskii* Stapf.  *Calligonum mongolicum* Turcz. | No  No  No  No | AM  AM  Non  AM | -25.26  -25.59  -23.69  -13.75 | 1.26  6.14  2.45  6.12 |  |
| Gedaxiang, Neimeng | 40°45´N  100°24´E | 1400 | 58.6 | Desert | *Reaumuria soongorica* (Pall.) Maxim.  *Nitraria sibirica* Pall.  *Ephedra przewalskii* Stapf. | No  No  No | AM  AM  Non | -25.58  -26.48  -27.01 | 0.18  5.87  0.63 |  |
| Shibanquan, Neimeng | 40°32´N  100°29´E | 1320 | 56.7 | Desert | *Reaumuria soongorica* (Pall.) Maxim.  *Nitraria sibirica* Pall.  *Ephedra przewalskii* Stapf.  *Hedysarum mongolicum* Turcz. | No  No  No  Yes | AM  AM  Non  AM | -24.84  -24.82  -26.83  -25.99 | 1.87  6.92  1.06  3.73 |  |
| Luogezhuang, Neimeng | 39°55´N  100°57´E | 1390 | 90.1 | Desert | *Nitraria sibirica* Pall.  *Calligonum mongolicum* Turcz. | No  No | AM  AM | -23.97  -14.26 | 6.7  1.61 |  |
| Yanchixiang, Neimeng | 39°47´N  101°13´E | 1340 | 94.6 | Saline land | *Tamarix ramosissima* Ledeb.  *Kalidium cuspidatum* (Ung.-Sternb.) Grub. | No  No | AM  Non | -27.74  -26.32 | 8.68  12.96 |  |
| Luochengxiang, Neimeng | 39°43´N  101°25´E | 1320 | 105.3 | Desert | *Reaumuria soongorica* (Pall.) Maxim.  *Nitraria sibirica* Pall.  *Ephedra przewalskii* Stapf. | No  Yes  No | AM  AM  Non | -27.92  -25.99  -25.4 | 3.32  4.01  1.0 |  |
| Shadan, Ningxia | 38°46´N  106°10´E | 1830 | 194.3 | Desert | *Hedysarum mongolicum* Turcz.  *Artemisia arenaria* DC.  *Sophora alopecuroides* L.  *Zygophyllum gobicum* Maxim. | Yes  No  Yes  No | AM  AM  AM  AM | -24.41  -26.31  -26.4  -24.5 | 4.63  1.72  2.66  1.71 |  |
| Tufosi, Ningxia | 38°17´N  106°06´E | 1870 | 205.9 | Shrubs | *Reaumuria soongorica* (Pall.) Maxim.  *Caragana microphylla* Laxm.  *Artemisia arenaria* DC.  *Artemisia anethoides* Mattf. | No  Yes  No  No | AM  AM  AM  AM | -25.68  -25.77  -25.08  -25.82 | -3.94  -1.67  -0.47  -0.79 |  |
| Jinchuan, Gansu | 38°41´N  102°18´E | 1420 | 138.6 | Desert | *Reaumuria soongorica* (Pall.) Maxim.  *Nitraria sibirica* Pall. | No  No | AM  AM | -25.13  -26.14 | 0.32  5.25 |  |
| Ayouqi, Neimeng | 39°10´N  101°44´E | 1580 | 116.6 | Desert | *Reaumuria soongorica* (Pall.) Maxim.  *Zygophyllum gobicum* Maxim. | No  No | AM  AM | -25.51  -23.01 | 0.52  3.7 |  |
| Mingqingcijingzi, Gansu | 39°01´N  101°59´E | 1400 | 124.5 | Desert | *Reaumuria soongorica* (Pall.) Maxim.  *Nitraria sibirica* Pall. | No  No | AM  AM | -24.13  -25.38 | 0.05  5.86 |  |
| Mingqingtaojiajing, Gansu | 38°57´N  102°16´E | 1340 | 121.4 | Desert | *Nitraria sibirica* Pall.  *Kalidium cuspidatum* (Ung.-Sternb.) Grub. | No  No | AM  AM | -26.35  -26.2 | 7.17  3.12 |  |
| Mingqinghongshagang, Gansu | 38°58´N  102°29´E | 1400 | 107.6 | Desert | *Reaumuria soongorica* (Pall.) Maxim.  *Nitraria sibirica* Pall.  *Salsola collina* Pall. | No  No  No | AM  AM  Non | -26.99  -26.17  -13.05 | 1.01  4.56  3.92 |  |
| Mingqinghongguozijing, Gansu | 38°55´N  102°43´E | 1380 | 106 | Desert | *Reaumuria soongorica* (Pall.) Maxim.  *Nitraria sibirica* Pall.  *Zygophyllum gobicum* Maxim. | No  No  No | AM  AM  AM | -26.59  -24.45  -26.59 | 0.32  3.96  0.32 |  |
| Mingqingxiaojingzi, Gansu | 38°50´N  102°54´E | 1350 | 105 | Desert | *Reaumuria soongorica* (Pall.) Maxim.  *Nitraria sibirica* Pall.  *Salsola ruthenica* Iijin.  *Peganum harmala* L. | No  No  No  No | AM  AM  Non  AM | -27.72  -27.51  -26.35  -26.06 | 1.57  2.89  -0.77  5.13 |  |
| Mingqinghongshaliangxiang, Gansu | 38°59´N  103°30´E | 1310 | 107.5 | Desert | *Lycium ruthenicum* Murr.  *Salsola laricifolia* Turcz. Ex Litv.  *Salsola passerina* Bge.  *Salsola collina* Pall. | No  No  No  No | AM  Non  Non  Non | -27.97  -26.83  -14.04  -14.12 | 7.22  6.41  3.1  3.94 |  |
| Huanghuatanxiang, Ningxia | 37°38´N  105°09´E | 1340 | 268.8 | Desert | *Artemisia annua* L.  *Artemisia roxburghiana* Bess.  *Sophora alopecuroides* L.  *Halogeton arachnoideus* Moq. | No  No  Yes  No | AM  AM  AM  Non | -25.2  -24.12  -25.81  -23.95 | -0.23  3.55  -0.07  2.98 |  |
| Shuangcao, Ningxia | 37°05´N  105°18´E | 1900 | 286.3 | Desert | *Reaumuria soongorica* (Pall.) Maxim.  *Nitraria tangutorum* Bobr.  *Peganum harmala* L.  *Zygophyllum gobicum* Maxim.  *Salsola collina* Pall.  *Salsola passerina* Bge. | No  No  No  No  No  No | AM  AM  AM  AM  Non  Non | -28.48  -25.67  -26.48  -28.72  -13.34  -14.51 | -0.19  5.33  1.19  -0.46  2.57  4.84 |  |
| Zhitanxiang, Ningxia | 37°31´N  105°39´E | 1810 | 254.1 | Desert | *Reaumuria soongorica* (Pall.) Maxim.  *Nitraria sibirica* Pall.  *Peganum harmala* L.  *Roegntria nutans* (Keng) Keng | No  No  No  No | AM  AM  AM  AM | -29.6  -27.98  -25.73  -25.23 | -0.03  2.57  4.13  -0.23 |  |

^*^ References:

1. Smith SE, Read DJ (2008) Mycorrhizal Symbiosis. New York: Academic Press.

2. Tian C, Shi Z, Chen Z, Feng G (2006) Arbuscular mycorrhizal associations in the Gurbantunggut Desert. Chinese Science Bulletin 51 Supp.: 140-146.
